# Efficacy of equine botulism antitoxin in botulism poisoning in a guinea pig model

**DOI:** 10.1371/journal.pone.0209019

**Published:** 2019-01-11

**Authors:** Andrew Emanuel, Hongyu Qiu, Douglas Barker, Teresa Takla, Karen Gillum, Nancy Neimuth, Shantha Kodihalli

**Affiliations:** 1 Research and Development, Emergent BioSolutions Canada Inc., Winnipeg, Manitoba, Canada; 2 Battelle Biomedical Research Center, West Jefferson, Columbus, Ohio, United States of America; CEA (Atomic and alternative energies commission), FRANCE

## Abstract

**Background:**

Botulism is a disease caused by neurogenic toxins that block acetylcholine release, resulting in potentially life threatening neuroparalysis. Seven distinct serotypes of botulinum neurotoxins (BoNTs) have been described and are found in nature world-wide. This, combined with ease of production, make BoNTs a significant bioweapon threat. An essential countermeasure to this threat is an antitoxin to remove circulating toxin. An antitoxin, tradename BAT (Botulism Antitoxin Heptavalent (A, B, C, D, E, F, G)–(Equine)), has been developed and its efficacy evaluated against all seven serotypes in guinea pigs.

**Methods and findings:**

Studies were conducted to establish the lethal dose and clinical course of intoxication for all seven toxins, and post-exposure prophylactic efficacy of BAT product. Animals were monitored for signs of intoxication and mortality for 14 days. Guinea pig intramuscular LD_50s_ (GPIMLD_50_) for all BoNTs ranged from 2.0 (serotype C) to 73.2 (serotype E) of mouse intraperitoneal LD_50_ units. A dose of 4x GPIMLD_50_ was identified as the appropriate toxin dose for use in subsequent efficacy and post-exposure prophylaxis studies. The main clinical signs observed included hind limb paralysis, weak limb, change in breathing rate/pattern, and forced abdominal respiration. Mean time to onset of clinical signs ranged from 12 hours (serotype E) to 39 hours (serotype G). Twelve hours post-intoxication was selected as the appropriate time point for intervention for all serotypes apart from E where 6 hours was selected because of the rapid onset and progression of clinical signs. Post-exposure treatment with BAT product resulted in a significantly (p<0.0001) higher survival at >0.008 scaled human dose for serotypes A, B, C, F and G, at >0.2x for serotype D and >0.04x for serotype E.

**Conclusions:**

These studies confirm the efficacy of BAT as a post-exposure prophylactic therapy against all seven known BoNT serotypes.

## Introduction

Botulism is a disease caused by toxins produced from spore forming anaerobic Gram-positive bacteria belonging to the genus *Clostridium* [1]. These botulinum neurotoxins (BoNTs) are extremely toxigenic, causing paralysis by blocking the release of acetylcholine predominantly at peripheral cholinergic nerve terminals of the skeletal and autonomic nervous system, with an estimated human lethal dose (LD_50_) extrapolated from animal species when the toxin is administered intravenously (IV), subcutaneously (SC), or, intraperitoneally (IP) of 1 ng/kg and 3 ng/kg by inhalation [1, 2]. Historically, all botulinum neurotoxin producing clostridia were grouped as *C*. *botulinum*. Today, at least six physiologically and genetically distinct bacteria are known to form BoNT, including *C*. *botulinum* Groups I-IV, some strains of *C*. *baratii*, C. *butyricum* and neurotoxin producing *C*. *sporogenes* [3]. Classification of botulinum neurotoxins (BoNTs) is based on toxin type and metabolic activity. Seven serologically distinct neurotoxic proteins, designated BoNT/A-G, distinguishable with animal antisera, have been described [1, 4, 5]. Genomic sequencing has confirmed the distinctiveness of these seven BoNT serotypes, with amino acid differences ranging from 37.2% to 69.6% [3]. *C*. *botulinum* Group I strains include BoNT/A and proteolytic strains of BoNT/B and/or BoNT/F and can produce multiple distinct neurotoxins. Group II strains include BoNT/B4 and nonproteolytic strains of BoNT/E or BoNT/F6 and are not known to produce multiple toxins. Group III includes nonproteolytic BoNT/C and BoNT/D and Group IV includes only strains that produce BoNT/G [3]. Group III strains primarily cause botulism in various animal species. Group IV strains have not currently been associated with either human or animal disease.

In humans, four serotypes (BoNT A, B, E and rarely F) are predominantly responsible for botulinum intoxication, but in the context of bioterrorism, any of the seven serotypes could be used. Serotype A and B strains have been identified worldwide, with the exception of Antarctica, and cause food-borne, infant, and wound botulism. Serotype A is responsible for the highest rate of mortality in humans. Serotype F strains share the same global distribution as serotypes A and B, but the incidence of botulism from BoNT/F is relatively rare by comparison. There is a single report published in 1981 of four adult deaths and one infant death caused by serotype G in Argentina [6]. In contrast, *C*. *botulinum* type E strains have a limited geographical distribution and occur primarily in northern countries such as Canada, Finland, Japan, Norway, Sweden, Russia, and Alaska in the United States [7]. Serotypes C, D and E cause illness in other mammals, birds and fish [8].

In the United States, a total of 205 confirmed and 10 probable cases of botulism were reported to CDC in 2016. Among confirmed cases, infant botulism accounted for 150 (73%) cases, foodborne botulism for 29 (14%) cases, wound botulism for 24 (12%) cases, and botulism of unknown transmission category for 3 (1%) cases. Among probable cases, foodborne botulism accounted for 8 cases and wound botulism for 2 cases [9]. In Europe, 201 suspected and 146 confirmed cases were reported in 2015, by a total of 18 European Union/European Economic Area (EU/EEA) countries with a notification rate of <0.1 cases per 100,000 population [10].

Our understanding of BoNTs has increased with advances in genomic sequencing. In 2014, Pellett *et al* isolated and cultured a bivalent strain from a 2014 clinical case of infant botulism and suggested the designation of serotype “H” [11]. After further analysis by the CDC this was designated as BoNT/FA. Recently, Zhang *et al* reported a novel serotype, BoNT/X, which was identified using genomic sequencing and bioinformatics [12] of *C*. *botulinum* strain 111. This serotype is not recognized by antisera against serotypes A to G [12]. Although similar to BoNT/B/D/F/G in that it cleaves to vesicle-associated membrane proteins (VAMP), it does so at a novel site. BoNT/X is also unique in that it also cleaves non-canonical substrates VAMP4, VAMP5 and Ykt6. Zhang and co-workers have also published a brief report of an isolate of *Enterococcus faecium* from a bovine source carrying a BoNT-like toxin designated BoNT/Eni [13]. The significance of this discovery is not yet known, but is of concern as *E*. *faecium* is a ubiquitous commensal, usually non-pathogenic in healthy individuals, but associated with nosocomial infections and the development of antibiotic resistance in the human population [14]. The current understanding of the biology, toxin production and mechanism of action of these toxins within nerve endings is reviewed in detail by Pirazzini and Rossetto [15]. The BoNT/X, BoNT/En may be hypothetical with no evidence of this serotype occurring in nature.

Botulinum neurotoxins (BoNTs) are considered to be some of the most toxic substances known, and have been classified as one of the six most potentially dangerous agents for bioterrorism and designated as category A, Tier 1 in the United States [16]. The rationale behind this designation is the extreme potency of the toxin, the relative ease with which it can be isolated and used with malice, and the severity of the clinical presentation of disease caused by the toxin [17]. It is worthwhile pointing out that the whereabouts of considerable quantities of BoNT produced by Iraq during the Gulf War are unknown [1]. An essential countermeasure against weaponized botulinum toxin is an effective vaccine; however, the development of such a vaccine is a complicated task. A formalin-inactivated toxoid against BoNT was tested as a vaccine in humans in the1930s [18], and a pentavalent BoNT vaccine received Investigational New Drug (IND) status for at-risk workers in the mid-1960s and for military deployment in 1991 by the United States Army Office of the Surgeon General IND 3723 [18]. In 2011, the Centers for Disease Control and Prevention (CDC) withdrew its recommendation for the use of the investigational pentavalent (A, B, C, D, E) vaccine for workers at risk of occupational exposure and changed the guidance for booster vaccination for military personnel only when a significant fall in antibody titers had occurred (rather than an annual schedule). These recommendations were made in response to a decline in the immunogenicity of the pentavalent vaccine against some serotypes and an increase in the number of local adverse reactions [19]. In addition to the low incidence of cases of botulism, distinct number of serotypes causing disease and identification of new previously unknown serotypes such as BoNT/FA and BoNT/X [11, 12], the number and variety of settings in which BoNT has clinical applications is particularly problematic for the development of an effective and safe vaccine for exposure to botulinum toxin, particularly in the event of its use as a chemical weapon [17]. Therefore, alternative treatments for botulism intoxication are of great value to the medical and defense communities.

BAT_,_a botulism antitoxin heptavalent drug product, was developed for the treatment of symptomatic botulism caused by serotypes A to G in adults and pediatric patients and licensed in the United States under 21 CFR Part 601 (Subpart H, Animal Rule), “Approval of Biological Products When Human Efficacy Studies Are Not Ethical or Feasible”. Under this rule, the BAT development program consisted of efficacy evaluation in two animal models and safety evaluation in healthy human volunteers.

BAT produce allows clearance of toxins through the liver and spleen, decreasing the availability of these toxins and preventing distribution to nerve endings [5]. BAT product is the only botulism antitoxin licensed for treatment of all seven known BoNT serotypes in both adult and pediatric patients. The efficacy of BAT product against serotype A (BoNT/A) in Rhesus macaques has previously been confirmed in a therapeutic and post-exposure prophylaxis study [20]. Due to ethical constraints with the use of a large number of non-human primates, the efficacy is confirmed against one serotype in Rhesus macaques.

A variety of parenteral routes can deliver the toxin, and these are the most straightforward exposure paradigm allowing for tight control of exposure parameters. Based on a comparative study with various routes of exposure, guinea pigs are most susceptible to exposure by IP route than an oral or inhalational route. The oral LD_50_ is about 70x to 5000x higher compared to IP LD_50_ depending on the toxin serotype [21]. Although, the oral and inhalational routes are the most likely routes for a potential bioweapons attack, the route of exposure is not relevant since the pathogenesis of BoNT is due to blood-borne toxemia. Aerosol and oral exposures are associated with high variability [22,23], making these routes of exposure less robust for the evaluation of a therapeutic intervention compared to parenteral routes of exposure. Therefore, for these studies, the IM route of exposure was selected to reduce variability in animals through more accurate exposure.

Here we report on three studies leading to the development of a guinea pig model and demonstration of the efficacy of BAT product against BoNT serotypes A, B, C, D, E, F and G in a post-exposure prophylactic setting. The goal of the initial study was to determine the LD_50_ for all seven serotypes for use in subsequent efficacy studies in guinea pig model. This was followed by definition of the clinical course of the disease in guinea pigs to monitor the progression of disease for the purpose of confirming challenge dose and determining the appropriate time of BAT intervention for each serotype in post-exposure prophylaxis (prior to onset of clinical signs) or therapeutic studies (definitive signs of systemic botulism). Lastly, the ability of the BAT product to enhance survival was demonstrated for all serotypes when administered at a fixed time point after exposure but prior to the onset of signs of intoxication.

## Methods

### Measurement of BoNT potency

The recognized measurement of BoNT potency has been defined as the median lethal dose of neurotoxin needed to cause 50% death of a population of Swiss Webster Mice (LD_50_) [24,25,26]. While LD_50_ values for serotypes A-E were established by Cardella in 1964, this had not previously been established for serotypes F and G [21]. The LD_50_ values for mice (serotypes A-E) range from 0.5 to 5 ng/kg. Potency may also be reported as LD_50_s per mg of toxin [27].

### Animals, animal husbandry and veterinary care

All studies were conducted at Battelle Biomedical Research Center. A total of 440 Hartley guinea pigs (*Cavia porcellus*) supplied by Charles River Laboratories (Kingston, NY, Raleigh, NC or St. Constant, Canada) were used. Only animals that were in good health, free of malformations, exhibiting no signs of clinical disease, and weighing between 400 and 500 g were used in these studies.

Husbandry was in accordance with test facility standard operating procedures for the care of rodents. All guinea pigs were housed in compliance with United States Department of Agriculture (USDA) guidelines [28]. Animals were individually housed in polycarbonate cages in stainless steel racks, equipped with automated watering systems and were on a 12-hour light/dark cycle (Study 1) or 24-hour continuous room lighting (Studies 2 and 3) in order to facilitate hourly and half-hourly observations of clinical signs of botulinum intoxication. All animals were provided with individual tinted huts as an optional shelter from continuous lighting. The bedding material utilized was Sani-chips hardwood heat-treated chips. Animals received both water and PMI Certified Guinea Pig Diet 5026 *ad libitum*. Housing room temperatures were maintained at 68 to 75°F and relative humidities were 23 to 58% while study animals were present. To enhance the psychological well-being of the animals and to provide optional shelter from continuous room lighting, each animal was provided with a tinted individual plexiglas “hut” within the cage. The huts were removed from cages after observation of the first severe clinical sign or if the shelter interfered with the animal’s mobility. Animals were identified by individual cage cards and ear tags.

No pain-relieving measures were used in the study as the use of analgesics may unintentionally change the outcome provided by the antibody treatment, thus, making it impossible to interpret the data obtained in this study. For euthanasia, anesthetics such as ketamine and xylazine hydrochloride were used to anesthetize animals before administering euthanasia agent Beuthanasia-D solution.

### Ethics statement

The research was conducted in compliance with the Animal Welfare Act (AWA, 7 U.S.C. §2131, 2002, 2007 and 2008) and other federal statutes and regulations relating to animals and experiments involving animals and adhered to the principles stated in the Guide for the Care and Use of Laboratory Animals [29]. All animal procedures were conducted under protocols approved by the Institutional Animal Care and Use Committees (IACUC) of Battelle Biomedical Research Center, in according with IACUC guidelines, https://www.nal.usda.gov/awic/institutional-animal-care-and-use-committees.

### Toxin

Partially purified (~95% purity) botulinum neurotoxin complexes (serotypes A through G) were used for the studies reported here. Serotypes A to E were produced at the University of Wisconsin and received at the test facility; serotypes F and G were produced at Metabiologics, Inc., Madison, Wisconsin, and received by the same facility (S1 Table). Serotypes A through F were received as ammonium sulfate precipitates and were reconstituted prior to use. Serotype G was received in phosphate buffered saline (PBS), pH 6.2. All botulinum neurotoxins were in Complex form. Serotypes E and G were produced as inactive forms and it was necessary to activate them using trypsin proteolytic treatment prior to intoxication. Toxin was administered as a single IM injection of a specific serotype (A to G) into the muscles of the right hind leg.

### Immunization, plasmapheresis and BAT production

BAT product is a hyperimmune plasma product prepared from horses that have been immunized with a specific serotype of commercially available botulinum toxoid (0.5% formalin inactivated, Metabiologics, Inc) or toxin to achieve high neutralizing antibody titers. Large quantities of plasma were collected using plasmapheresis. Following plasmapheresis, the immune globulin fraction was purified using a validated manufacturing process. This process for each antitoxin type includes cation-exchange chromatography to purify the immune globulin fraction, digestion with pepsin to produce F (ab') 2 plus F (ab') 2-related immune globulin fragments, anion exchange chromatography to remove the pepsin as well as other impurities and filtration. Removal of Fc using pepsin minimizes the potential for immunogenic reactions with equine products in humans.

In addition, the manufacturing process includes two viral inactivation/removal steps; solvent/detergent (S/D) treatment and virus filtration. The S/D treatment step is effective at inactivating known lipid-enveloped viruses such as equine encephalitis, equine arteritis, West Nile virus, equine infectious anemia, equine herpes virus, rabies, and equine influenza. The BAT manufacturing process also includes a robust filtration step that is effective in reducing the levels of some lipid-enveloped viruses as well as non-enveloped viruses including equine rhinovirus, equine adenoviruses and equine parvovirus [20].Following formulation, the individual botulism antitoxins are blended into the final heptavalent product. The blended BAT lot (Lot # 2060401X) used in these studies contained a total protein concentration of 56mg/mL, placebo contained 50mg/mL (Lot #107034800) total protein. The antitoxin potency was determined using an *in vivo* neutralization assay. The potency and stability of BAT product used in animal studies were confirmed. Seven vials of BAT (Botulism Antitoxin Heptavalent (A, B, C, D, E, F, G)–(Equine)) Lot #2060401X manufactured by Emergent BioSolutions (Winnipeg, Canada) were used in the Study 3. One vial was used for each of the seven serotype phases (S2 Table).

### Phosphate buffered saline control, placebo

For definition of clinical course of BoNT intoxication the Control Material was 0.2% gelatin phosphate buffer, purchased from Northeast Laboratory Services (Winslow, ME 04901, USA). This buffer was prepared by Northeast Laboratory Services according to the master formula provided by Battelle, and stored at 2–8°C.

In Study 3, Control Groups received Placebo (Lot #10703480) manufactured by Emergent BioSolutions (Winnipeg, Canada). Botulism Antitoxin Placebo (normal equine immune globulin), was manufactured using a similar process to the manufacture of BAT described above including the digestion with pepsin to produce F (ab') 2 plus F (ab') 2-related immune globulin fragments. Placebo had a protein concentration of 50 mg/ml and potency of *<*0.38 Units/vial against all seven BoNT.

The test article and control article dilution material used was normal saline (0.9% sodium chloride USP).

### Euthanasia criteria

For all studies, humane endpoints were established to minimize or terminate the pain or distress via euthanasia. The following pre-established criteria were followed for euthanasia:

1. Any guinea pig judged to be moribund (prostrate or unresponsive) by a trained technician, Study Director or Study Veterinarian was euthanized after the first scheduled observation.
2. Any guinea pig having a ≥20% weight loss in combination with severe clinical signs (described in S3 Table) were euthanized.

On study Day 14 or 21 (depending on the study) surviving animals were euthanized after the completion of the study. Animals requiring euthanasia were deeply anesthetized with ketamine (35 to 40 mg/animal) and xylazine (6 to 7mg/animal) IM and then euthanized with 0.5 to 1.0 mL intracardiac Beuthanasia-D solution.

### Design of studies

#### Study 1—Lethal dose ranging study of botulinum neurotoxin complex (BoNT) serotypes A, B, C, D, E, F, G

The objective of this study was to determine the guinea pig intramuscular lethal dose 50% (GPIMLD_50_) of botulinum neurotoxin complex (BoNT) serotypes A to G in male Hartley guinea pigs for subsequent use in selecting a toxin challenge dose in subsequent efficacy studies. Prior to the initiation of the study, the potency of each BoNT serotype was determined by mouse assay (in male CD-1 (ICR) mice) and expressed as MIPLD_50_ (Mouse Intraperitoneal Lethal Dose 50%) units/mL. Guinea pig challenge dose dilution calculations were based on mouse potency assay results.

A range of BoNT dilutions (0.2x to 5.0x) around the expected historical LD_50_ were administered to naïve male guinea pigs by a single IM injection for serotypes A-E. The historical LD_50_s, as noted previously, were only available for five of the seven serotypes [21]. A range of BoNT dilutions from 10 to 1,000 MIPLD_50_ units were administered to naïve male guinea pigs by IM injection as a screening stage for serotypes F and G. A subsequent second stage for serotypes F and G utilized a range of dilutions centered around the estimated GPIMLD_50_ calculated from Stage 1. Dose levels for serotypes F and G were selected based on an estimated GPIMLD_50_ values. All challenged guinea pigs were examined twice daily at intervals of at least 6 hours apart for signs of intoxication and mortality for 14 days.

#### Study 2—Confirmatory neurotoxin challenge dose and clinical course studies

The primary objectives of Study 2 were to (a) confirm guinea pig mortality following intramuscular (IM) injection of a challenge dose of Botulism Toxin (BoNT) at 4x GPIMLD_50_ for each toxin serotype (A through G) determined in Study 1; and (b) to conduct an extensive evaluation of the clinical signs of intoxication, in order to determine the time to onset of clinical signs, duration of clinical signs and time to death for each serotype to develop criteria to statistically predict early signs of intoxication for intervention in subsequent therapeutic studies.

Toxin preparations of 4x GPIMLD_50_ were prepared for each serotype and were administered to naïve male and female guinea pigs via a single intramuscular (IM) injection. A Control Group was administered dose preparation buffer for comparison purposes. Toxin preparations (except for serotype E) were assigned an identification code for blinding purpose. After receipt, but prior to study Day 0, all animals determined to be suitable for study were randomized by weight using SAS plan (version 9.1.3) to gender-balanced study groups and subsequently identified only by number, to ensure technical staff were blinded to the serotype administered and remove any potential bias in recording clinical observations. Animals were caged individually. Time of intoxication and clinical observations relative to intoxication were also recorded for all animals.

Animals were monitored for mortality and clinical signs of intoxication for up to 14 days. On study Days 0 to 3, all groups (except serotype E) were monitored hourly; from study Day 4 until study Day 14, the surviving (Control Group) animals were monitored twice daily. Serotype E animals were monitored every 30 minutes until death because of rapid onset and progression of the clinical disease with this serotype.

#### Study 3—Post-exposure intravenous dose ranging study

The objective of this study was to assess the post-exposure prophylactic (PEP) efficacy of BAT product in terms of survival when administered following an intramuscular botulism neurotoxin (BoNT) intoxication equivalent to 4x GPIMLD_50_ of Serotypes A to G. The efficacy study was designed to test the null hypothesis that there would be a similar survival rate at the end of the study in the BAT treated and placebo treated animals. In order to determine the minimum effective prophylactic dose, BAT product was administered by IV prior to the onset of clinical signs, at a range of doses (1x, 0.2x, 0.04x, 0.008x scaled human dose) or placebo approximately 12 hours after toxin administration for Serotypes A, B, C, D, F, G and approximately 6 hours post intoxication for Serotype E. Based on the assumption that 1 vial of BAT product (containing 11.17 mL at 56 mg/mL of protein) is one scaled human dose, and assuming that the average human weighs 70 kg, the dose volume per unit of body weight would be equivalent to 0.16 mL/kg (11.17 mL/70 kg). Assuming the nominal dose level to be administered is 0.16 mL/kg and assuming a target dose volume of 2 mL/kg (equivalent to 1 mL per 500 g animal) the appropriate dilution factor (total target volume / BAT volume) was calculated to be 12.5. This dilution resulted in a dose formulation with a protein concentration of 4.48 mg/mL. The time-points selected were generated from Study 2 and were expected to occur prior to the onset of clinical signs of intoxication. For each of the seven serotype phases, animals were randomized to five groups of 10 males and 10 females per group.

All animals were monitored twice daily during quarantine including prior to Day 0 intoxication. Following IM administration of BoNT, trained technicians observed the animals, beginning within 3.5 hours of the first animal’s time of toxin injection for each phase. Bodyweight measurements were taken on days 4, 7, 14 and 21 post-intoxication. Loss in body weight of greater than 20% along with sever signs of intoxications were used as criteria for euthanasia. After the initial observation, on study Days 0–6 clinical observations were performed approximately hourly (day and night) on all animals except those intoxicated with serotype E, which had observations performed approximately every 30 minutes (day and night). From study Day 7 until termination on study Day 21, animals were monitored approximately every three hours if showing moderate or severe clinical signs, or twice daily if showing only mild or no clinical signs.

### Statistical analysis

Analyses were conducted prior to each study to provide statistical justification for the sample size used. Survival data were analyzed to determine the GPIMLD_50_ for each toxin type using probit dose-response models [30]. Fieller’s method or the delta method [31] was used to compute 95 percent confidence intervals for the GPIMLD_50_ values. For the serotypes in which confidence intervals could not be computed using probit analysis (when the probit slope was infinitely steep and could not be accurately estimated), the Spearman-Karber method [32] was used.

The mean time to signs of intoxication and mean time to death were calculated using regression models suitable for right-censored data. Clinical signs included in the statistical analysis for Study 2 and 3 are shown in S3 Table. Survival proportions were compared using Fisher’s exact test with the Bonferroni Holm adjustment for multiple comparisons. The Kaplan-Meier method was used for the analysis of the time to onset of clinical signs and the time to death, with comparisons between groups done using the log rand test with the Bonferroni Holm adjustment for multiple comparisons. Nonparametric survival analysis, the mean duration of each clinical sign, mean duration of any clinical signs, and the mean duration of lethargy plus hind limb local paralysis/weak limbs were calculated using SAS (SAS Institute, Cary, NC).

## Results

### Study 1—GPIMLD_50_ determination

Thirty-five (35) guinea pigs were used for each serotype A, B, C, D, F, and G; fifty (50) guinea pigs were used for serotype E. The same lot and batch number of each serotype from the mouse potency assays was used in the study. Sample size was determined using simulation modeling (SAS v 9.1.3) and was sufficient to estimate LD_50_ to within 2-fold of the historical LD_50_ (21), with a 97% probability of estimating a confidence interval.

Median lethal dose at 1x GPIMLD_50_ across the seven serotypes ranged from 2.0 (serotype C) to 73.2 (serotype E) MIPLD_50_ units (Table 1). Table 2 shows the mean time to predicted onset of clinical signs of intoxication and mortality at 1x and 4x GPIMLD_50._

**Table 1:** Median lethal dose (1x GPIMLD_50_ listed in MIPLD_50_ units) for male guinea pigs 400-500g.

Serotype	GPIMLD_50_ (MIPLD_50_ units per guinea pig)	95% Confidence Interval (MIPLD_50_ units)
A	4.5	(3.2, 6.6)^1^
B	8.5	(7.4, 9.8)^2^
C	2.0	(1.2, 2.6) ^1^
D	5.7	(4.9, 6.5)^2^
E	73.2	(66.3, 86.4)^1^
F	25.0	(20.8, 30.0) ^2^
G	53.2	(48.7, 58.2)^2^

^1^. The probit slope was statistically significant for serotypes A, C and E

^2^. The probit slope was infinitely steep and could not be accurately estimated for serotypes B, D, F and G; therefore, the Spearman-Karber method was used to calculate the GPIMLD50 and the 95 percent confidence intervals.

**Table 2:** Predicted Mean Time to onset of clinical signs and mean time to death of male guinea pigs at 1x and 4x GPIMLD_50_ by BoNT Serotype.

Serotype	Predicted Mean Time to Onset of Clinical Signs (Days)		Predicted Mean Time to Mortality (Days)	
	1x GPIMLD_50_	4x GPIMLD_50_	1x GPIMLD_50_	4x GPIMLD_50_
A	4.5	1.5	11.4	2.7
B	3.7	1.3	11.6	2.5
C	3.6	1.0	12.3	3.4
D	3.0	1.2	9.8	2.9
E	2.0	0.8	10.2	0.8
F	6.6	1.5	8.2	2.0
G	4.8	1.5	9.7	2.6

The most commonly observed clinical signs were hind limb (local) paralysis, lethargy and weak limbs. Serotypes A, B, C and D had greater total numbers of clinical signs than serotypes E, F and G. A dose related increase in the incidence and severity of clinical signs was noted for all serotypes, however, some higher dose animals died while only presenting mild/minimal clinical signs and some animals in serotypes E, F and G were found dead without presenting any prior clinical signs. Because the observations recorded every six hours, it is possible animals showed signs and died between observation cycles.

**Fig 1:**
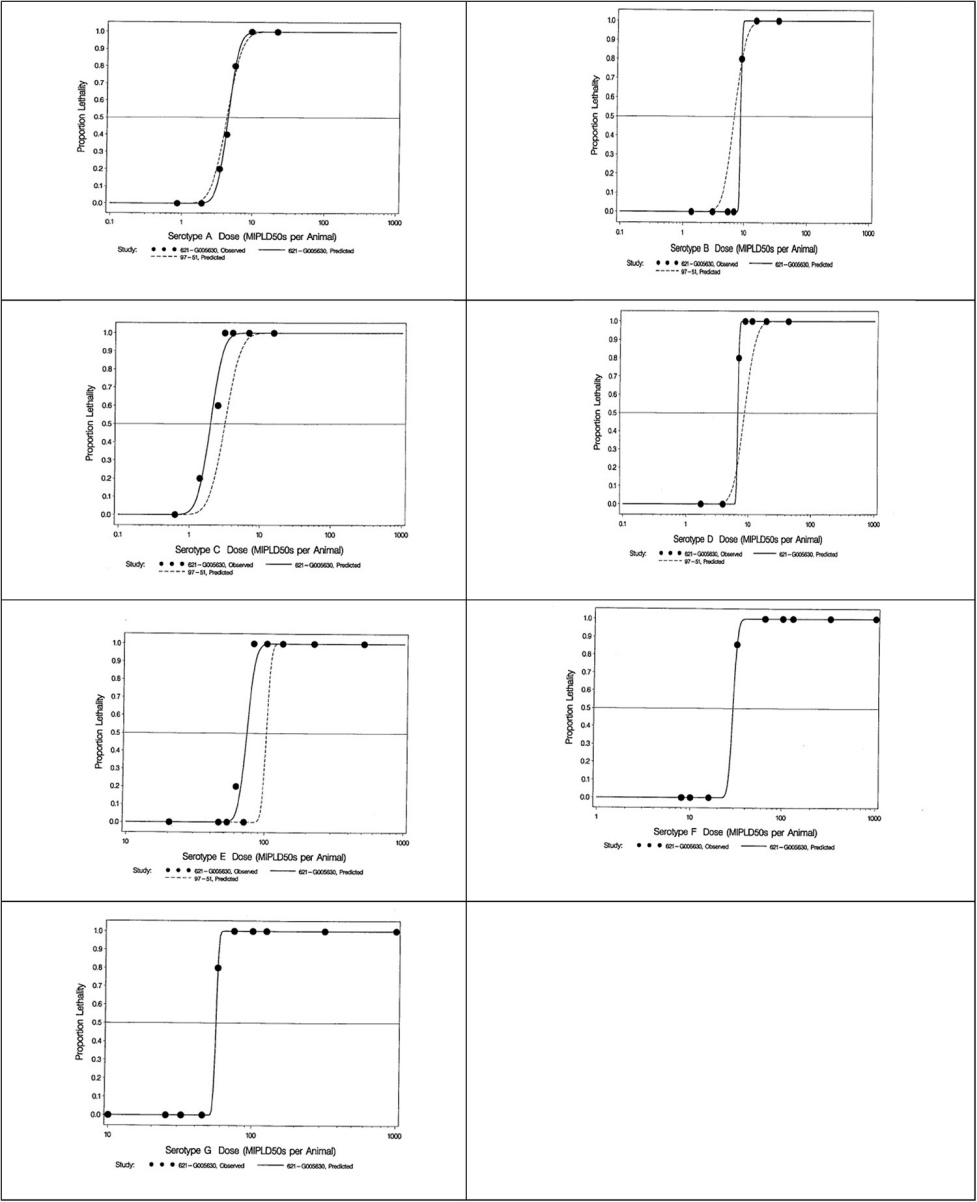
Probit dose response graphs for serotypes A to G (IM). Mortality data (black circles) were plotted and Probit dose response models (solid lines) were fitted to the proportion of lethality as a function of the base -10 logarithm of toxin dose for each serotype. Historical data (dotted lines) was available for all serotypes except BoNT/F and BoNT/G and is overlaid for comparative purposes.

Using the mortality data slopes, probit dose response models were fitted to the proportion lethality as a function of the base -10 logarithm of toxin dose for each serotype. The probit dose response graphs for all seven serotypes is shown in Fig 1. The GPIMLD_50_ for serotypes A to E were similar to historical LD_50_ data from Cardella [21]. In addition, the GPIMLD_50_ for serotypes F and G were newly established.

4x GPIMLD_50_ was selected as the appropriate toxin dose for use in subsequent efficacy studies as it was expected to result in 100% mortality of untreated animals and the clinical course (time from onset of clinical signs through to death) would be of a reasonable duration to allow for intervention with BAT to result in recovery of intoxicated animals.

### Study 2—Clinical course

Eighty (40 male and 40 female) guinea pigs were assigned equally to 8 treatment groups (serotypes A to G and a Control). Animal numbers used were sufficient assuming a mortality rate of 90% and a lower 95% confidence bound greater than 50%, indicating that the level of mortality was unlikely to have occurred by chance. All BoNT-intoxicated guinea pigs died, confirming the lethality of the selected dose level for each serotype. All control animals survived until Day 14.

**Fig 2:**
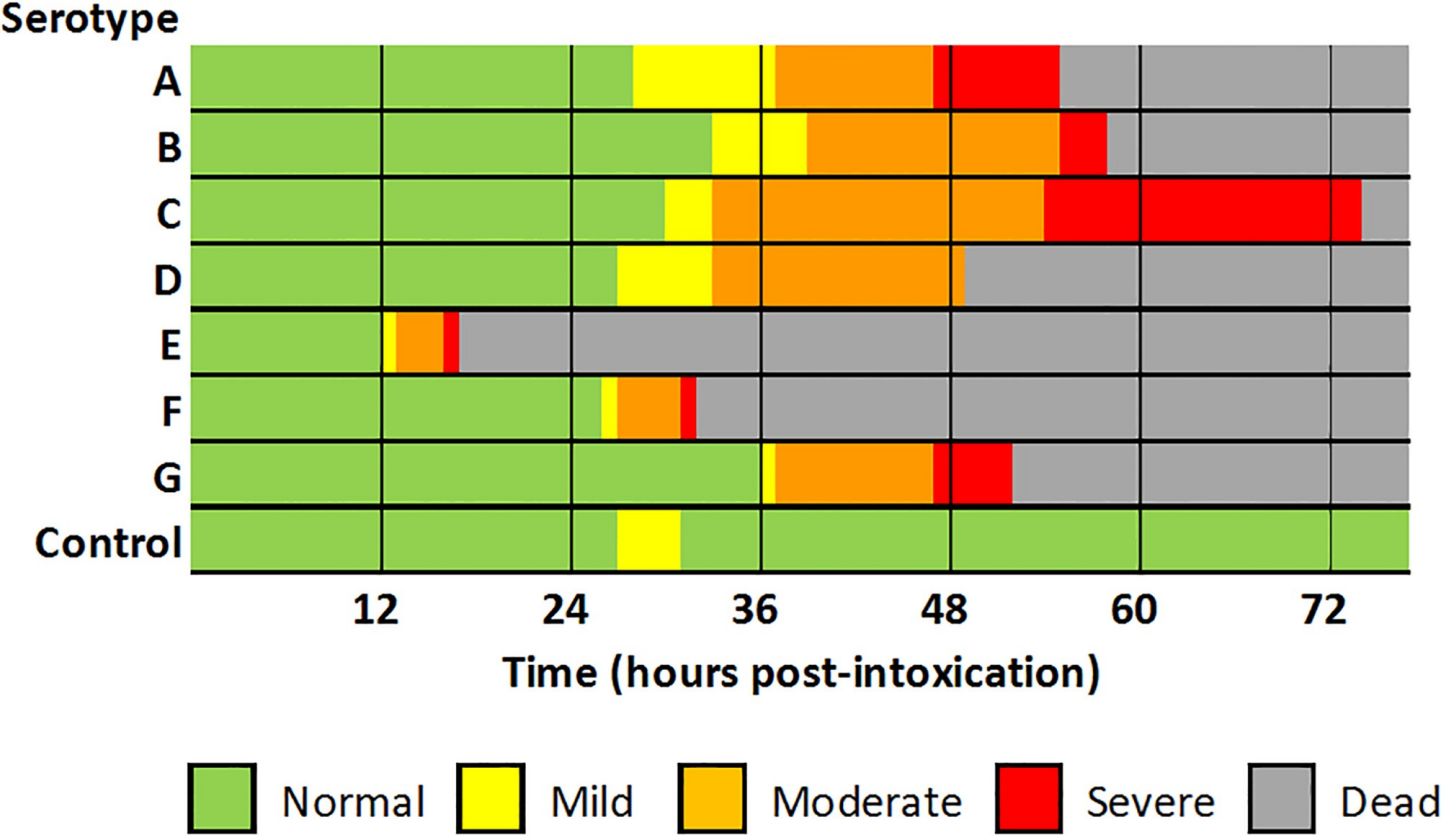
Mean time to onset of mild, moderate and severe signs of botulism intoxication and mean time to death for each of seven. BoNT serotypes. Mild signs: Lethargy. Moderate signs: Hind limb paralysis/weak limbs, Salivation, Lacrimation, Cannot rise, Noticeable change in breathing pattern/rate. Severe signs: Forced abdominal respirations and Total paralysis. Time to onset and duration of clinical signs varied between serotypes, but intoxication was universally lethal. A single animal in the buffer control group exhibited lethargy for ~ 4 hours.

Lethargy, hind limb paralysis and weak limbs were identified as being clinically relevant to botulism intoxication and progression due to the frequency and consistency across serotypes. Table 3 summarizes mean time to onset of relevant signs and time to death and corresponding time ranges. Clinical progression of botulism intoxication varied by serotype, with BoNT/E and BoNT/F showing the most rapid progression from mild signs of intoxication to death, and BoNT/C showing the slowest progression (Fig 2). Lethargy and hind limb local paralysis/weak limbs occurred in all but one intoxicated animal regardless of serotype and are therefore considered reliable and important early indicators of BoNT intoxication from any serotype (Table 4). The observation of transient lethargy in a single control animal can be attributed to the impact of frequent (hourly) observations on the animal (Table 4 and Fig 2). A number of BoNT-challenged animals exhibited non-protocol listed clinical signs of which emaciation (23/70, 32.86%) and audible breathing sounds or trembling (9/70, 12.86%) were the most commonly reported.

**Table 3:** Time to onset of clinically relevant early signs of intoxication and time to death.

	Mean Hours (Range) to Onset of Clinically Relevant Early Signs of BoNT Intoxication		
BoNT Serotype	Lethargy	Hind Limb Local Paralysis/Weak Limbs	Mean Hours (Range) to Death
A	28 (25, 39)	32 (26, 46)	55 (36, 73)
B	33 (25, 51)	39 (26, 47)	58 (46,76)
C	30 (25, 45)	33 (26, 42)	74 (55, 84)
D	27 (25, 31)	33 (26, 46)	48 (33, 62)
E	12 (10, 15)	13 (11, 14)	17 (12, 20)
F	26 (23, 31)	27 (23, 31)	32 (26, 45)
G	36 (26, 43)	39 (31, 41)	52 (34, 71)
Control	27 (27, 27)	NA	NA

**Table 4:** Number of animals per BoNT serotype showing selected clinical signs.

Clinical Sign	BoNT Serotype							
	A	B	C	D	E	F	G	Control
**Mild**								
Lethargy	10	10	10	10	10	10	9	1
**Moderate**								
Hind limb Local Paralysis/Weak Limbs	10	10	10	10	10	10	9	0
Salivation	8	6	8	4	0	1	9	0
Lacrimation	4	4	8	1	0	0	4	0
Noticeable Change in Breathing Pattern or Rate	10	10	10	9	9	9	6	0
**Severe**								
Forced Abdominal Respirations	10	7	10	7	8	8	8	0
Unable to Rise	8	9	10	8	6	1	3	0
Total Paralysis	4	3	2	2	0	1	0	0
**Other**								
Any Clinical Sign	10	10	10	10	10	10	9	1
Lethargy Plus Hind Limb Local Paralysis/Weak Limbs	10	10	10	10	10	10	9	0

One BoNT intoxicated animal in serotype G died prior to any clinical sign of intoxication.

Lethality of the 4x GPIMLD_50_ intoxication dose was confirmed. The trigger for therapeutic efficacy intervention was identified as the onset of the first moderate clinical sign (hind limb paralysis/weak limbs) for all seven serotypes. Twelve hours post-intoxication was selected for intervention for serotypes A to D, F and G in a post-exposure setting for Study 3, while 6 hours post-intoxication was selected for serotype E due to the rapid onset and progression of clinical signs for this serotype.

### Study 3—Post-exposure intravenous dose ranging study

For each of the 7 serotypes, 100 animals were randomized to five groups of 10 males and 10 females per group. Animal numbers were sufficient to reject the null hypothesis (no difference in survival rates between treatment and control groups) with 90% power, when the probability of survival is 70% in the treated group and 10% in the control group. The assumed survival rate of treated animals was based on data collected in a preliminary study of 22 animals (data not shown).

**Fig 3:**
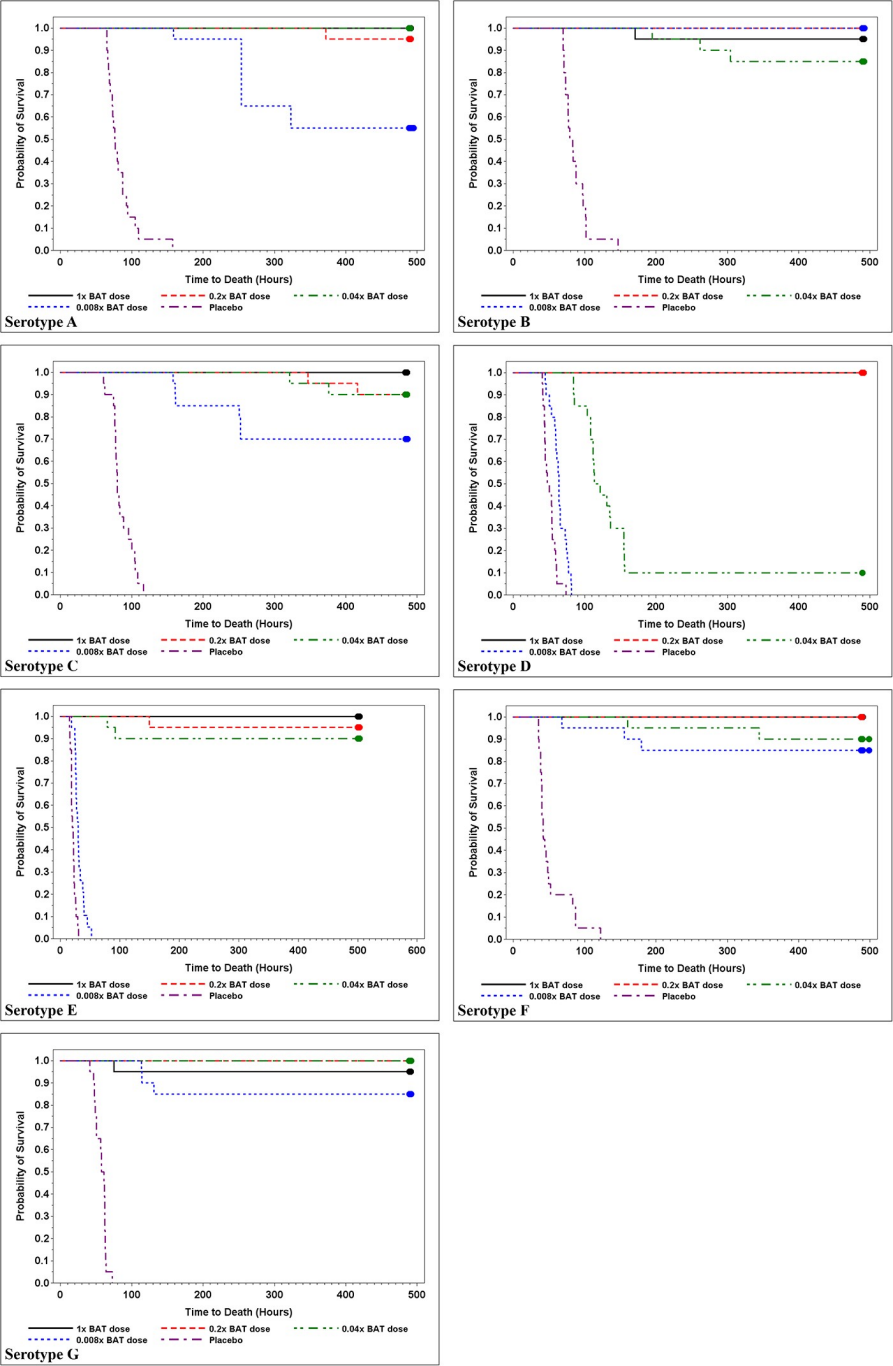
Kaplan-Meier survival graphs for serotypes A to G. Survival data for each of four BAT dose dilutions and placebo control is plotted against time for each of the seven serotypes tested. A significant decrease in mortality rates was observed among most BAT treated groups compared to the control group across all serotypes. Survival time, as represented by median time to death, was significantly greater than controls for all BAT antitoxin serotypes and dose dilutions tested.

Control groups in all of the 7 serotypes had 100% mortality, confirming the lethality of the toxin dose at 4 x GPIMLD_50._ (Table 5). A significant decrease in mortality was observed among most BAT treated animals compared to the control group. A statistically significant (p<0.001) improvement in survival rate was observed in BAT treated animals at all dose levels (≥0.008x scaled human dose) for serotype A, B, C, F, and G, and at ≥0.2x for serotype D, and ≥0.04x for serotype E. The median time to death in the BAT treated groups at all dose levels were significantly longer (log-rank test with Bonferroni-Holm adjustment p<0.0001) for all doses and all study groups (Table 5 and Fig 3). The disease progression in control animals from clinical onset to severe clinical signs, and eventually death, clearly indicated that in the absence of post-exposure prophylaxis, intoxication results in death. The dose response observed in the treated groups demonstrated the efficacy of prophylactic treatment against all seven serotypes at doses as low as 0.2x scaled human dose.

**Table 5:** Summary of guinea pig survival, time to death and time to onset of moderate clinical signs.

Serotype	Treatment Group (BAT)^a^	Survival Rate (%) and Fishers Exact Test Bonferroni-Holm Adjusted p-value Compared to Placebo Control	Kaplan-Meier Median Time to Death (95% Cl), in Hours, and Log-Rank Test Adjusted p-value Compared to Placebo Control	Kaplan-Meier Median Time to Onset of Any Moderate Clinical Sign (95% CI), in Hours
**A**	1x	20/20 (100%) p<0.0001*	— p<0.0001*	—
	0.2x	19/20^b^ (95%) p<0.0001*	— p<0.0001*	—
	0.04x	20/20 (100%) p<0.0001*	— p<0.0001*	— (123, —)
	0.008x	11/20 (55%) p = 0.0009*	— (254, —) p<0.0001*	108 (98, 116)
	Placebo Control	0/20 (0%) N/A	70 (70, 87) N/A	48 (45, 60)
**B**	1x	19/20 (95%) p<0.0001*	— p<0.0001*	— (101, —)
	0.2x	20/20 (100%) p<0.0001*	— p<0.0001*	—
	0.04x	17/20 (85%) p<0.0001*	— p<0.0001*	— (292, —)
	0.008x	20/20 (100%) p<0.0001*	— p<0.0001*	95 (66, 101)
	Placebo Control	0/20 (0%) N/A	81 (74, 97) N/A	55 (51, 56)
**C**	1x	20/20 (100%) p<0.0001*	— p<0.0001*	—
	0.2x	18/20^c^ (90%) p<0.0001*	— p<0.0001*	—
	0.04x	18/20^d^ (90%) p<0.0001*	— p<0.0001*	—
	0.008x	14/20 (70%) p<0.0001*	— (253, —) p<0.0001*	74 (73, 76)
	Placebo Control	0/20 (0%)N/A	80 (77, 96) N/A	45 (43, 47)
**D**	1x	20/20 (100%) p<0.0001*	— p<0.0001*	—
	0.2x	20/20 (100%) p<0.0001*	— p<0.0001*	— (109, —)
	0.04x	2/20 (10%) p = 0.9744	118 (108, 155) p<0.0001*	65 (60, 74)
	0.008x	0/20 (0%) p = 1.0000	64 (59, 73) p<0.0001*	38 (34, 49)
	Placebo Control	0/20 (0%) N/A	49 (44, 55) N/A	32 (31, 35)
**E**	1x	19/19^e^ (100%) p<0.0001*	— p<0.0001*	17 (16, 20)
	0.2x	19/20 (95%) p<0.0001*	— p<0.0001*	18 (16, 112)
	0.04x	18/20 (90%) p<0.0001*	— p<0.0001*	16 (16, 19)
	0.008x	0/19^f^ (0%) p = 1.0000	30 (26, 33) p<0.0001*	16 (15, 17)
	Placebo Control	0/20 (0%) N/A	21 (18, 23) N/A	13 (13, 15)
**F**	1x	20/20 (100%) p<0.0001*	— p<0.0001*	—
	0.2x	20/20 (100%) p<0.0001*	— p<0.0001*	—
	0.04x	18/20 (90%) p<0.0001*	— p<0.0001*	—
	0.008x	17/20 (85%) p<0.0001*	— p<0.0001*	54 (38, 63)
	Placebo Control	0/20 (0%) N/A	42 (39, 49) N/A	33 (32, 36)
**G**	1x	19/20 (95%) p<0.0001*	— p<0.0001*	—
	0.2x	20/20 (100%) p<0.0001*	— p<0.0001*	—
	0.04x	20/20 (100%) p<0.0001*	— p<0.0001*	—
	0.008x	17/20 (85%) p<0.0001*	—p<0.0001*	69 (66, 77)
	Placebo Control	0/20 (0%)	59 (50, 62)N/A	38 (38, 44)

^a^ Dose levels compared to proposed human dose (1 vial = human dose/70 kg body weight) scaled volume/kg basis Either the clinical sign was not observed, or the Kaplan-Meier estimates could not be calculated due to censoring. N/A = Not Applicable. * Comparison significant at the Bonferroni-Holm adjusted 0.05 level of significance.

^b^: One animal which succumbed lacked clinical sign progression typical of botulinum neurotoxin intoxication, but this death was attributed to botulism intoxication for purposes of data analysis.

^c^: Two animals which succumbed did not show prior clinical signs of botulinum neurotoxin intoxication, but these deaths were attributed to botulism intoxication for purposes of data analysis.

^d^: One animal which succumbed did not show clinical signs of botulinum neurotoxin intoxication for the 48 hours preceding death. A second animal which succumbed did not show prior clinical signs of botulinum neurotoxin intoxication. Both deaths were attributed to botulism intoxication for purposes of data analysis.

^e^: One animal excluded from analysis was euthanized due to hind-limb fracture.

^f^: One animal excluded from analysis removed from study due to incorrect randomization and resulting inappropriate dosing.

*: Statistically significant difference compared to placebo control

Control animals intoxicated with BoNT/A/B/C/F suffered from severe weight loss compared to baseline resulting in death or euthanasia within day 4 or 5 post-intoxication. Weight loss data for control animals intoxicated with BoNT/D/E/F were not available as all had succumbed before day 4. The body weight loss in treatment groups was dose-dependent. For BoNT/A/B/C/F/G, a BAT dose of higher than 0.04x resulted in body weight increase compared to baseline on all study days (Days 4, 7, 14, and 21). For these serotypes, the lowest dose (0.008x) reduced overall weight loss in animals that survived, and the recovery was slow except for animals intoxicated with BoNT/A. Animals intoxicated with BoNT/E showed consistent weight gain with only high dose treatment (1x and 0.2x). Treatment with a BAT dose of 0.04x resulted in substantial weight loss by Day 4 and a slow recovery above baseline after day 14 post-intoxication. Animals treated with the lowest dose (0.008x) succumbed before day 4. For BoNT/ D, only animals treated with the highest dose (1x) showed consistent weight gain after intoxication. Animals treated with 0.2x dose levels demonstrated weight loss to between day 4 and 14, after which they started to gain weight. A few animals from 0.04x dose group survived with this serotype. As was the case with BoNT/E, BoNT/D animals treated with a BAT dose of 0.008x succumbed before Day 4 (Table 5, and S1 Fig).

A total of six animals died during the study without signs of typical BoNT intoxication. One animal from the group intoxicated with BoNT/A treated with 0.2x dose was found dead on day six post-intoxication. Two animals from 0.2x dose group and two from 0.0.04x dose group exposed to BoNT/C were found dead between 14 and 18 days post-intoxication. One animal from the 0.04x dose group intoxicated with BoNT/F was found dead on day 15 post-intoxication. All these six animals showed no clinical signs consistent with BoNT intoxication immediately before death. The cause of death was not determined as no post-mortem examinations were performed. As per the protocol and pre-specified analysis plan, all these animals were included in the analysis. One animal from BoNT/ E group treated with 1x BAT dose was euthanized due to accidental trauma (broken leg) on 12 days post-intoxication and excluded from the study. One animal from BoNT/E group treated with 0.008x BAT dose was also excluded from analysis due to inappropriate dosing. Statistical evaluation of mortality data was performed and the dose of BAT antitoxin that would result in 80% survival for each serotype was calculated. The lower confidence interval (based on 95% confidence intervals calculated by Fieller’s method) of this calculated dose was identified as the Minimum Effective Dose (Table 6). BAT antitoxin protected against toxin serotypes A-G when administered as a post-exposure prophylactic treatment. The minimum effective dose of BAT antitoxin which was found to give at least 80% survival in guinea pigs intoxicated with any of the seven serotypes (A to G) was determined to be 0.078x scaled human dose. Although, less than 1x dose was efficacious in post-exposure setting, it was assumed that a higher dose of antitoxin to immediately counter the toxin effects in a treatment scenario would be needed.

**Table 6:** Minimum effective dose (MED) of BAT antitoxin per Serotype.

Serotype	A	B	C	D	E	F	G
MED BAT product*	0.027x	0.054x	0.052x	0.078x	0.039x	0.013x	0.051x

* Compared to proposed clinical BAT dose (1x) on a volume/kg basis, 11.17 mL (1 vial) per 70 kg.

## Discussion

Prior to the conduct of these studies, no established animal models existed for use in botulism clinical or efficacy studies for all seven BoNT serotypes (A to G). In addition, the Animal Rule had never been used to seek regulatory approval of a multivalent antitoxin product. The development of a robust guinea pig model in the three studies reported here determined toxin potency, characterized the clinical profile/onset and progression of the disease and demonstrated the efficacy of BAT in a post-exposure prophylaxis setting. The requirement to assess the efficacy of BAT product against each of the seven serotypes independently necessitated the use of a guinea pig model as other models (i.e. rat and rabbit) have been shown to be insensitive to one or more toxin serotypes [33]. Due to ethical reasons, it was not possible to use non-human primates for all seven serotypes, though they are sensitive to all seven serotypes of toxins. Guinea pigs were selected for the study because of their use in other studies investigating other equine-derived botulinum antitoxins [34] and (like humans) are sensitive to multiple toxin serotypes [21, 35, 36]. While the primary disease of botulism (progressive paralysis resulting in death) is comparable between the guinea pig, Rhesus macaques and humans, specific details differ between the species. In the guinea pig, there is an early onset of clinical signs of intoxication, specifically muscular weakness and respiratory distress, then a slow progression as these conditions worsen, eventually resulting in paralysis and death, although the time of this progression varies by serotype. The typical early manifestations of the disease in Rhesus macaques include ptosis, dysphagia, muscular weakness, respiratory distress and total paralysis (20). The general progression of the intoxication in guinea pigs is comparable to signs observed in Rhesus macaques and consistent with symptoms from reported human cases (2). Despite the comparable disease profile in animals and humans, there are differences among species and limitations of the models compared with the human clinical setting. Nevertheless, the guinea pig model has shown to be a robust, reproducible and a good substitute for evaluation of BAT against all seven serotypes.

The LD_50_ for all seven serotypes was confirmed and, as previously reported, the toxin dose was inversely related to the duration of clinical course [22, 37]. The clinical profile of the disease is consistent across serotype suggesting the suitability of the model for efficacy evaluation of antitoxins against all seven serotypes. For serotype E and G, there was a significant difference in the MIPLD_50_ units required to achieve 1x GPIMLD_50_ in comparison to other serotypes. This toxicity data is consistent with the previous findings suggesting that the serotype E is less toxic in guinea pigs compared to other serotypes (A, B, C, D) [21]. Although less toxic, the disease onset with serotype E is rapid due to its unique structure resulting in a faster rate of intoxication than any other serotype [38]. No historical information on sensitivity is available for serotype G.

This is the first report where a therapeutic has tested and successfully demonstrated efficacy against all seven botulinum serotypes in a relevant model. Antibodies obtained from the plasma of hyper-immunized hosts are effective only against toxins circulating in body fluids. BAT product is indicated for the treatment of symptomatic botulism following documented or suspected exposure to botulinum neurotoxin serotypes A, B, C, D, E, F, or G in both adults and pediatric patients. In order to demonstrate the efficacy of BAT product by improving the survival of treated animals, it was necessary to establish the effect of a range of toxin doses on clinical progression and mortality. In this series of studies, the common response to toxin was a progressive muscular paralysis starting with limb weakness and respiratory distress. This increased in severity until animals were moribund due to respiratory or muscular paralysis. Study 3 confirmed the post-exposure prophylactic efficacy of BAT product in preventing the occurrence/progression of clinical signs of BoNT intoxication and death when administered prior to the onset of clinical signs across all seven serotypes (A to G). The minimum effective dose giving 80% survival across all seven serotypes was determined to be 0.078x scaled human dose. The control animals displayed significant clinical signs, and eventually death and demonstrated that in the absence of prophylactic BAT treatment, intoxication results in severe disease and eventual death.

The reduced efficacy observed for BoNT/D could be due to the low level of specific antitoxin component in the heptavalent product (1452 Units/vial), however, it is important to note that similar levels of serotype G antitoxin (1229 Units/vial) provided better survival at lower doses (85% survival at 0.008x BAT dose) in comparison to BoNT/D (0% survival at 0.008x BAT dose). The neutralization capacity of one human dose of BAT product is well above the amount required to neutralize the highest documented BoNT exposure levels reported for humans. The highest serum levels of BoNT reported in the US is 32 MIPLD_50_/mL; this corresponds to a total body load of 480,000 MIPLD_50_ [39]. The highest serum level of BoNT ever reported in the world is 160 MIPLD_50_/mL; this corresponds to a total body load of 2,400,000 MIPLD_50_ [40]. Based on the standard neutralization capacity of one unit of antitoxin, in theory, a BAT dose can neutralize 6 million MIPLD_50_ of BoNT/D and about 5 million MIPLD_50_ of BoNT/E. However, several factors including the antibody dose, toxin dose, and timing of administration can affect the efficacy following intoxication [22, 23, 41]. The ratios of antitoxin to toxin required for successful toxin neutralization vary depending on the toxin dose/serotype. Studies have indicated that equine antitoxins are most effective with antitoxin/toxin ratios of at least 30:1 and ratios required is higher (100:1) with lower toxin challenge [42]. It is possible that actual antitoxin/toxin ratios played a role in the observed efficacy against serotype D and E toxins. Studies in humans have suggested the association between early administration of antitoxin and improved survival, length of hospital stay and use of ventilation [41, 43]. For serotype D and E, antitoxin-toxin ratios along with the timing of administration may have influenced the efficacy at lower dose levels. Time and dose-ranging studies are required to pinpoint factors affecting the effectiveness of BAT against these toxin serotypes.

Currently, BAT product is the only approved intervention for the treatment of symptomatic botulism following documented or suspected exposure to botulinum neurotoxin serotypes A, B, C, D, E, F, G in adult and pediatric patients. The results from the studies in a guinea pig model confirm the effectiveness of BAT antitoxin against all seven serotypes as a post-exposure prophylactic agent at doses as low as 0.2x scaled human dose.

Recently, a number of new serotypes have been identified. Barash and Arnon published a report of a novel toxin “type H” [44], however this was reclassified as BoNT/FA in 2016 [11]. BoNT/FA is neutralized by available antitoxins, supporting this reclassification. In 2017 another new BoNT was reported and tentatively named BoNT/X [12]. BoNT/X has a unique substrate profile, and of particular concern is that this novel toxin is not recognized by antisera against known BoNTs. It remains unknown whether BoNT/X is ever produced outside the laboratory in *C*. *botulinum*. The same group of researchers also reported an *Enterococcus faecium* from cow feces carrying a BoNT-like toxin, designated BoNT/En [13]. This is the first report of BoNT-like gene clusters outside of *Clostridium* and is of particular concern because of the prevalence of this organism in the human population and its association with antibiotic resistance. With the continuing advances in genomics it is likely that additional toxins will be identified in the coming years, although to date there is limited evidence of these serotypes in nature and they are mainly hypothetical proteins. The significance of these new discoveries remains to be seen.

In addition to neutralizing the effects of toxins by immunization, other opportunities for developing effective therapies include monoclonal antibodies; inhibitors of toxin binding, toxin internalization and trafficking or toxin translocation; inhibitors of SNARE cleavage; and the possibility of developing therapies that effect the reversal of BoNT paralysis. The effectiveness of the guinea pig model described here in demonstrating the efficacy of BAT product as a post-exposure prophylactic treatment of botulinum intoxication shows that this model will be a cornerstone for the development of future agents.

These studies confirm the efficacy of BAT product as a post-exposure prophylactic therapy against all seven serotypes and add important data to our knowledge of the effect of intoxication with different botulinum toxins and the progression of clinical signs and mortality in guinea pig models of the disease. Notwithstanding the potential for the use of BoNTs as agents for bioterrorism, the development of an effective therapy is also essential to counter the possible use of these toxins in military theaters. These studies suggest that BAT product is an effective option to control botulism in such situations prior to the development of physical and clinical signs of intoxication.

**Trade marks**: Emergent BioSolutions, Protected by Emergent BioSolutions, BAT, and any and all Emergent BioSolutions Inc. brand, product, service and feature names, logos and slogans are trademarks or registered trademarks of Emergent BioSolutions Inc. or its subsidiaries in the United States or other countries. All other brand, product, service and feature names or trademarks are the property of their respective owners.

## Supporting information

S1 Table Potency of botulism antitoxin and capacity to neutralize maximum known serum concentration of toxin reported in the literature based on the label claim. (DOCX)

S2 Table Botulinum neurotoxin serotype and lot number. (DOCX)

S3 Table Classification of common clinical signs and severity^1^. (DOCX)

S1 Fig Observed percent body weight changes by serotype and treatment group on day 4, 7, 14 and 21 post-intoxication. Guinea pigs were intoxicated with 4xLD50 of botulinum toxin serotypes A, B, C, D, E, F or G and subsequently treated with 1x (hollow blue circles), 0.2x (orange circles), 0.04x (hollow black triangles) or 0.008x (yellow triangles) BAT or placebo (x). Body weights were taken 4, 7, 14- and 21-days post-intoxication and compared with baseline (Day 0) body weights to determine percent weight changes.
(TIF)

## References

[1] Anderson J, Hilmas CJ. Chapter 28—Botulinum Toxin* A2—Gupta, Ramesh C. Handbook of Toxicology of Chemical Warfare Agents (Second Edition). Boston: Academic Press; 2015 p. 361–85.

[2] Middlebrook JL, Franz DR. Botulinum Toxins. Medical Aspects of Clinical and Biological Warfare. http://www.globalsecurity.org1997. p. 643–54.

[3] Peck MW, Smith TJ, Anniballi F, Austin JW, Bano L, Bradshaw M, et al Historical Perspectives and Guidelines for Botulinum Neurotoxin Subtype Nomenclature. Toxins. 2017;9(1). 10.3390/toxins9010038 . 28106761 PMC5308270

[4] Dorner MB, Schulz Km Fau—Kull S, Kull S Fau—Dorner BG, Dorner BG. Complexity of botulinum neurotoxins: challenges for detection technology. Curr Top Microbiol Immunol. 2013;364(0070-217X (Print)):219–55. 10.1007/978-3-642-33570-9_11 23239356

[5] Pirazzini M, Rossetto O, Eleopra R, Montecucco C. Botulinum Neurotoxins: Biology, Pharmacology, and Toxicology. Pharmacological reviews. 2017;69(2):200–35. Epub 2017/03/31. 10.1124/pr.116.012658 . 28356439 PMC5394922

[6] Sonnabend O, Sonnabend W, Heinzle R, Sigrist T, Dimhofer R, Krech U. Isolation of Clostridium botulinum Type G and identification of Type G botulinal toxin in humans: Report of five sudden unexpected deaths. J Inf Dis. 1981;143(1):22–7. 7012244 10.1093/infdis/143.1.22

[7] Macdonald TE, Helma CH, Shou Y, Valdez YE, Ticknor LO, Foley BT, et al Analysis of Clostridium botulinum serotype E strains by using multilocus sequence typing, amplified fragment length polymorphism, variable-number tandem-repeat analysis, and botulinum neurotoxin gene sequencing. Applied and environmental microbiology. 2011;77(24):8625–34. Epub 2011/10/18. 10.1128/AEM.05155-11 . 22003031 PMC3233090

[8] Hansbauer EM, Skiba M, Endermann T, Weisemann J, Stern D, Dorner MB, et al Detection, differentiation, and identification of botulinum neurotoxin serotypes C, CD, D, and DC by highly specific immunoassays and mass spectrometry. The Analyst. 2016;141(18):5281–97. Epub 2016/06/30. 10.1039/c6an00693k . 27353114

[9] Centers for Disease Control. National Botulism Surveillance Summary 2016, 20 February 2018. https://www.cdc.gov/nationalsurveillance/pdfs/botulism_cste_2015.pdf.

[10] European Centre for Disease Prevention and Control. Botulism—Annual Epidemiologic Report 2018 (2015 data)2018 21 August 2018. https://ecdc.europa.eu/sites/portal/files/documents/AER_for_2015-botulism.pdf.

[11] Pellett S, Tepp WH, Bradshaw M, Kalb SR, Dykes JK, Lin G, et al Purification and Characterization of Botulinum Neurotoxin FA from a Genetically Modified Clostridium botulinum Strain. mSphere. 2016;1(1). Epub 2016/06/16. 10.1128/mSphere.00100-15 . 27303710 PMC4863619

[12] Zhang S, Masuyer G, Zhang J, Shen Y, Lundin D, Henriksson L, et al Identification and characterization of a novel botulinum neurotoxin. Nature communications. 2017;8:14130 Epub 2017/08/05. 10.1038/ncomms14130 . 28770820 PMC5543303

[13] Zhang S, Lebreton F, Mansfield MJ, Miyashita SI, Zhang J, Schwartzman JA, et al Identification of a Botulinum Neurotoxin-like Toxin in a Commensal Strain of Enterococcus faecium. Cell host & microbe. 2018;23(2):169–76.e6. Epub 2018/02/06. 10.1016/j.chom.2017.12.018 . 29396040 PMC5926203

[14] Gao W, Howden BP, Stinear TP. Evolution of virulence in Enterococcus faecium, a hospital-adapted opportunistic pathogen. Curr Opin Microbiol. 2018;41:76–82. Epub 2017/12/12. 10.1016/j.mib.2017.11.030 . 29227922

[15] Pirazzini M, Rossetto O. Challenges in searching for therapeutics against Botulinum Neurotoxins. Expert opinion on drug discovery. 2017;12(5):497–510. Epub 2017/03/09. 10.1080/17460441.2017.1303476 . 28271909

[16] Rusnak JM, Smith LA. Botulinum neurotoxin vaccines: Past history and recent developments. Human vaccines. 2009;5(12):794–805. Epub 2009/08/18. . 19684478 10.4161/hv.9420

[17] Simpson LL. Botulinum Toxin In: Barrett ADT, Stanberry LR, editors. Vaccines for Biodefense and Emerging and Neglected Diseases: Academic Press; 2009 p. 891–917.

[18] Smith LA, Rusnak JM. Botulinum neurotoxin vaccines: past, present, and future. Critical reviews in immunology. 2007;27(4):303–18. Epub 2008/01/17. . 18197811 10.1615/critrevimmunol.v27.i4.20

[19] Notice of CDC's discontinuation of investigational pentavalent (ABCDE) botulinum toxoid vaccine for workers at risk for occupational exposure to botulinum toxins. MMWR Morbidity and mortality weekly report. 2011;60(42):1454–5. Epub 2011/10/28. . 22031218

[20] Kodihalli S, Emanuel A, Takla T, Hua Y, Hobbs C, LeClaire R, et al Therapeutic efficacy of equine botulism antitoxin in Rhesus macaques. PLoS ONE. 2017;12(11):e0186892 10.1371/journal.pone.0186892 29166654 PMC5699824

[21] Cardella MA. Botulinum Toxoids In: Lewis KH, editor. Botulism: Proceedings of a Symposium. PHS Publication: Washington DC: Government Printing Office; 1964 p. 113–30.

[22] Al-Saleem FH, Nasser Z, Olson RM, Cao L, Simpson LL. Identification of the factors that govern the ability of therapeutic antibodies to provide postchallenge protection against botulinum toxin: a model for assessing postchallenge efficacy of medical countermeasures against agents of bioterrorism and biological warfare. J Pharmacol Exp Ther. 2011;338(2):503–17. 10.1124/jpet.111.180653 . 21586604 PMC3141897

[23] Cheng LW, Henderson TD 2nd Comparison of oral toxicological properties of botulinum neurotoxin serotypes A and B. Toxicon. 2011; 58(1): 62–67. 10.1016/j.toxicon.2011.05.003. 21600236 PMC3124623

[24] McLellan K, Das RE, Ekong TA, Sesardic D. Therapeutic botulinum type A toxin: factors affecting potency. Toxicon: official journal of the International Society on Toxinology. 1996;34(9):975–85. . 8896190 10.1016/0041-0101(96)00070-0

[25] Sesardic D, Das RE, Corbel MJ. Botulinum toxin. J R Soc Med. 1994;87(5):307 . 8207732 PMC1294535

[26] Sesardic D, Jones RG, Leung T, Alsop T, Tierney R. Detection of antibodies against botulinum toxins. Mov Disord. 2004;19 Suppl 8:S85–91. 10.1002/mds.20021 . 15027059

[27] Peng Chen Z, Morris JG Jr., Rodriguez RL, Shukla AW, Tapia-Nunez J, Okun MS. Emerging opportunities for serotypes of botulinum neurotoxins. Toxins. 2012;4(11):1196–222. 10.3390/toxins4111196 . 23202312 PMC3509704

[28] Animal Care Policy Manual. United States Departed of Agriculture; 2007.

[29] National Research Council. Guide for the Care and Use of Laboratory Animals. 7th Edition ed: National Academic Press; 1996.

[30] Finney DJ. Probit Analysis. New York, NY: Cambridge University Press; 1971 333 p.

[31] Nelson W. Applied Life Data Analysis. New York, NY: Wiley; 1982.

[32] Hamilton MA, Russo RC, Thurston RV. Trimmed Spearman-Karber method for estimating median lethal concentrations in toxicity bioassays. Environmental Science & Technology. 1977;11(7):714–9. 10.1021/es60130a004

[33] Greenbaum SB, Anderson JB, Lebeda FJ. Botulinum Toxins In: Swearengen JR, editor. Biodefense Research Methodology and Animal Models2012. p. 415.

[34] Metzger JF, Lewis GE Jr., Human-derived immune globulins for the treatment of botulism. Rev Infect Dis. 1979;1(4):689–92. Epub 1979/07/01. . 399376 10.1093/clinids/1.4.689

[35] Ciccarelli AS, Whaley DN, McCroskey LM, Gimenez DF, Dowell VR Jr., Hatheway CL. Cultural and physiological characteristics of Clostridium botulinum type G and the susceptibility of certain animals to its toxin. Applied and environmental microbiology. 1977;34(6):843–8. . 74236 10.1128/aem.34.6.843-848.1977 PMC242758

[36] Harvey SM, Sturgeon J, Dassey DE. Botulism due to Clostridium baratii type F toxin. J Clin Microbiol. 2002;40(6):2260–2. 10.1128/JCM.40.6.2260-2262.2002 . 12037104 PMC130751

[37] Iida H, Ono T, Karashimada T. Experimental studies on the serum therapy of type E botulism: the relationship between the amount of toxin in the blood and the effect of antitoxic serum. Jpn J Med Sci Biol. 1970;23(5):344–7. Epub 2011/05/19 1970/10/01.. 4993667

[38] Kumaran D, Eswaramoorthy S, Furey W, Navaza J, Sax M, Swaminathan S. Domain organization in Clostridium botulinum neurotoxin type E is unique: its implication in faster translocation. J Mol Biol. 2009;386(1):233–45. 10.1016/j.jmb.2008.12.027 . 19118561

[39] Hatheway CH, Snyder JD, Seals JE, Edell TA, Lewis GE Jr., Antitoxin levels in botulism patients treated with trivalent equine botulism antitoxin to toxin types A, B, and E. J Infect Dis. 1984;150(3):407–12. . 6481185 10.1093/infdis/150.3.407

[40] Ball AP, Hopkinson RB, Farrell ID, Hutchison JG, Paul R, Watson RD, et al Human botulism caused by Clostridium botulinum type E: the Birmingham outbreak. Q J Med. 1979;48(191):473–91. . 575566

[41] Tacket CO, Shandera WX, Mann JM, Hargrett NT, Blake PA. Equine antitoxin use and other factors that predict outcome in type foodborne botulism. Am J Med. 1984; 76: 794–798. 6720725 10.1016/0002-9343(84)90988-4

[42] Sheridan RE, Deshpande SS, Amersdorfer P, Marks JD, Smith T. Anomalous enhancement of botulinum toxin type A neurotoxicity in the presence of antitoxin. Toxicon: official journal of the International Society on Toxinology. 2001;39(5):651–7. . 11072043 10.1016/s0041-0101(00)00189-6

[43] McCarty CL, Angelo K, Beer KD, White KC, Quinn K, Fijter S, Bokayani R et al Notes from the Field: Large Outbreak of Botulism Associated with a Church Potluck MealÐOhio, 2015Weekly. 7 31, 2015; 64(29): 802–803 10.15585/mmwr.mm6429a6 PMC4584836 26225479

[44] Barash JR, Arnon SS. A novel strain of Clostridium botulinum that produces type B and type H botulinum toxins. J Infect Dis. 2014; 209(2):183–191 10.1093/infdis/jit449 24106296

