# Supplementary material for: Efficacy of equine botulism antitoxin in botulism poisoning in a guinea pig model
Source: PLoS One. 2019 Jan 11;14(1):e0209019. doi: 10.1371/journal.pone.0209019 (PMC6329499; doi:10.1371/journal.pone.0209019)
Supplement: S1 Table — (DOCX) [file pone.0209019.s001.docx]

| **Serotype** | **Label Claim U/vial (Target Potency U/vial)^a^** | **Toxin Neutralization Capacity Based on Label Claim (MIPLD_50_)** | **Theoretical number of times antitoxin**  **could neutralize BoNT levels** | |
| --- | --- | --- | --- | --- |
|  |  |  | **480,000 MIPLD50**  **(US ^b^)** | **2,400,000 MIPLD50**  **(World ^c^)** |
| A | >4500 (10399) | 45,000,000 | 195.3 | 39.1 |
| B | >3,300 (7071) | 33,000,000 | 143.2 | 28.6 |
| C | >3000 (7138) | 30,000,000 | 130.2 | 26.0 |
| D | >600 (1452) | 6,000,000 | 26.0 | 5.2 |
| E | >5100 (10690) | 5,100,000 | 22.1 | 4.4 |
| F | >3000 (6546) | 30,000,000 | 130.2 | 26.0 |
| G | >600 (1229) | 6,000,000 | 26.0 | 5.2 |

^a^Blended lot 2060401X, fill volume 11.17 mL/vial; **^b^**Hatheway et al (39); ^c^ Ball et al. (40). U = Unit; US = United States. Each unit of antitoxin against serotype A, B, C, D, F, and G neutralize 10,000 MIPLD_50_ of respective toxins, while each unit of
