# Supplementary material for: Efficacy of equine botulism antitoxin in botulism poisoning in a guinea pig model
Source: PLoS One. 2019 Jan 11;14(1):e0209019. doi: 10.1371/journal.pone.0209019 (PMC6329499; doi:10.1371/journal.pone.0209019)
Supplement: S2 Table — (DOCX) [file pone.0209019.s002.docx]

| **Serotype** | **Lot number** |
| --- | --- |
| A | A01195 |
| B | B01195 |
| C | C012495 |
| D | D022505 |
| E | E011295 |
| F | F033001-01 |
| G | G092905-01 |
