# Supplementary material for: Efficacy of equine botulism antitoxin in botulism poisoning in a guinea pig model
Source: PLoS One. 2019 Jan 11;14(1):e0209019. doi: 10.1371/journal.pone.0209019 (PMC6329499; doi:10.1371/journal.pone.0209019)
Supplement: S3 Table — (DOCX) [file pone.0209019.s003.docx]

| Severity | Clinical Sign | Description |
| --- | --- | --- |
| Mild  Mild clinical signs are frequently observed and are probably due to botulism intoxication, but could not be considered a definitive indicator of the disease. | Lethargy | Decreased alertness or activity level; indifference or excessive drowsiness. |
| Moderate  Moderate clinical signs are unequivocally indicative of botulism intoxication and appeared in the early/mid stages of the disease | Salivation (Sal) | Presence of excessive salivation, or drooling, causing wetness outside of the mouth, such as on the muzzle or chin |
|  | Lacrimation (Lac) | Excessive lacrimation is an overproduction of tears, causing wetness around the eye(s), or pooling of tears at eyelid edges or at the corners of the eye(s) |
|  | Hind Limb Local Paralysis | Slowness, abnormal position, dragging, trembling, or awkward movement of the BoNT-injected hind leg only |
|  | Weak Limb (WL) | Weakness of multiple legs; may be slow, awkward upon movement, may be held in an abnormal position, or may drag, tremble, splay outward or otherwise be associated with an abnormal gait when the animal moves |
|  | Noticeable change in breathing sounds or pattern (ChBr) | Any change in breathing sounds or pattern; audible breathing sounds such as rasps, squeaks, whistles or chirps, excessively deep breathing, excessively shallow breathing, unusually slow or rapid breathing, and/or persistent irregular breathing |
| Severe  Severe clinical signs are extremely debilitating and occur late in the disease progression, shortly before death. | Forced abdominal respirations (FAR) | Exaggerated, deep spasmodic movement of the flank (abdominal wall) muscles during inspiration |
|  | Total paralysis (TP) | Loss of purposeful (effective) voluntary movement |

^1. Ruffled fur was omitted from the analysis^
