# Supplementary figures and images for: Efficacy of equine botulism antitoxin in botulism poisoning in a guinea pig model
Source: PLoS One. 2019 Jan 11;14(1):e0209019. doi: 10.1371/journal.pone.0209019 (PMC6329499; doi:10.1371/journal.pone.0209019)

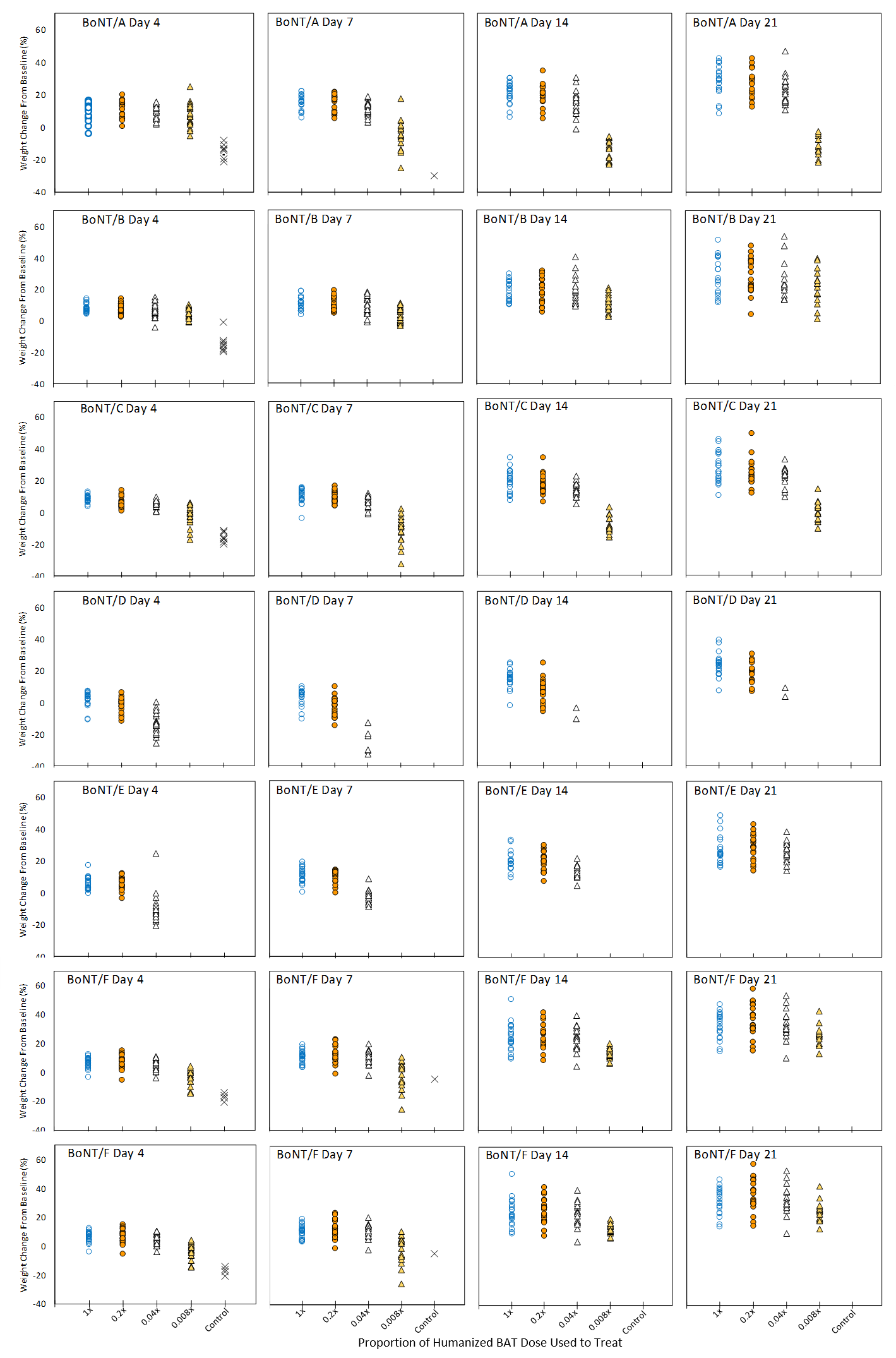

Supplement: S1 Fig — Guinea pigs were intoxicated with 4xLD50 of botulinum toxin serotypes A, B, C, D, E, F or G and subsequently treated with 1x (hollow blue circles), 0.2x (orange circles), 0.04x (hollow black triangles) or 0.008x (yellow triangles) BAT or placebo (x). Body weights were taken 4, 7, 14- and 21-days post-intoxication and compared with baseline (Day 0) body weights to determine percent weight changes. (TIF) [file pone.0209019.s004.tif]
